# Functional Analysis of Spike from SARS-CoV-2 Variants Reveals the Role of Distinct Mutations in Neutralization Potential and Viral Infectivity

**DOI:** 10.3390/v14040803

**Published:** 2022-04-13

**Authors:** Alona Kuzmina, Seraj Wattad, Stanislav Engel, Elli Rosenberg, Ran Taube

**Affiliations:** 1 The Shraga Segal Department of Microbiology Immunology and Genetics, Faculty of Health Sciences, Ben-Gurion University of the Negev, Beer Sheva 84105, Israel; kuzmina@post.bgu.ac.il (A.K.); serajw@post.bgu.ac.il (S.W.); 2 Department of Clinical Biochemistry and Pharmacology, Faculty of Health Sciences, Ben-Gurion University of the Negev, Beer Sheva 84105, Israel; engels@exchange.bgu.ac.il; 3 Soroka Medical Center, Beer Sheva 84105, Israel; eliros@bgu.ac.il

**Keywords:** COVID-19, SARS-CoV-2, spike, Pfizer–BTN162b2 vaccine, neutralization antibodies, furin–cleavage site, Receptor Binding Domain (RBD), SARS-CoV-2 variants, Delta, Lota

## Abstract

Enhanced viral transmission and escape from vaccine–elicited neutralizing antibodies drive worldwide spread of SARS-CoV-2 variants and promote disease progression. However, the impact of specific spike mutations that are carried by different viral variants on viral infectivity and neutralization sensitivity has not been completely defined. Here, we use pseudoviruses to assess the contribution of spike mutations within the Receptor Binding Domain (RBD) and the Furin Cleavage Site (FCS), and appear in circulating viral variants, on viral infectivity and neutralization potential against sera that was drawn from fully vaccinated individuals. Our functional analysis demonstrates that single, P681H, P681R or A701V–FCS mutations do not play a role in viral infectivity and neutralization potential. However, when in conjunction with the RBD–N501Y mutation, viral infectivity is enhanced. Similarly, combining the E484K–RBD mutation to the spike that carries FCS mutations reduces neutralization sensitivity with no effects on viral infectivity. Employing a similar approach onto the spike from Delta or Lota SARS-CoV-2 variants further reveals that specific RBD mutations affect neutralization sensitivity or viral infectivity differently. Our results validate the efficacy of the Pfizer third dose vaccine against Delta and Lota SARS-CoV-2 variants, and outline the significance of distinct RBD mutations in promoting viral infectivity and neutralization sensitivity to post–vaccination sera.

## 1. Introduction

In late 2019, an emergent betacoronavirus, Severe Acute Respiratory Syndrome Coronavirus 2 (SARS-CoV-2), was identified as the cause for severe respiratory COVID-19 disease in humans. One year into this outbreak, it is clear that the pandemic is here to stay, leading to approximately 4% mortality and causing global effects on both economic and quality of life [1,2]. Efforts to minimize spread of the pandemic led to a rapid development and authorization of several vaccines, among them mRNA vaccines—Pfizer (BNT162b2) and Moderna (mRNA1273)—that target the Receptor Binding Domain (RBD) of the original Wuhan viral spike. These vaccination efforts have successfully enhanced both humoral and cellular immune protection against viral infections, providing broad protection against infection by circulating variants of concern and severe disease progression [3,4,5,6,7,8,9,10,11,12,13,14]. Nevertheless, rapid spread and high rates of viral evolution led to the emergence of new variants that display enhanced transmission and partly challenge vaccine efficacy. This process is driven by both escape from neutralizing antibodies, where mutations like E484K, E484Q, S477N play key roles, and increased binding affinity of spike to the receptor, N501Y, T478K, K417N, and D614G are important. The combination of these mutations within variants of concern, mainly Beta, Delta, and Omicron, has substantially promoted viral spread and evolution combined with decrees in vaccine efficiency [15,16,17,18,19,20,21,22].

The viral spike (S) glycoprotein is a trimeric class I fusion transmembrane glycoprotein with S1 and S2 subunits that are non–covalently bound. Following attachment of S1 subunit to ACE2 receptor, two steps of proteolytic cleavage are executed; one at the S1/S2 boundary and the other at the S2′ site, which promotes membrane fusion between envelopes of the virus and its cell target. A polybasic _681_PRRAR/SV_687_ cleavage site in SARS-CoV-2 spike at the S1/S2 boundary serves as a Furin Cleavage Site (FCS) has been suggested to increase viral transmissibility by facilitating maturation of the spike precursor through furin-like proteases in producer cells prior to virion release [23]. Following this step, TMPRSS2 on the target cell surface and cathepsins B and L in endosomes cleave the S2′ site to promote membrane fusion [24,25].

While the involvement of mutations within the Receptor Binding Domain (RBD) of spike are extensively being tested for effects on antibody-neutralization and viral infectivity, the role of _681_PRRAR/SV_687_ FCS mutations—P681H found in Alpha or Omicron, V701A in Beta or P681R in Delta—in affecting neutralization and infectivity levels have not been explored. This polybasic motif in spike has recently been shown to enhance membrane fusion and syncytia formation [26,27,28,29]. It also facilitates TMPRSS2 endocytic viral entry and promotes IFITM2 innate immune evasion [30]. Removal of this motif significantly reduces (but does not abolish) viral spike processing [24,31,32,33,34,35,36,37,38], and, in addition, renders viral entry highly pH and cathepsin dependent in late endosomes [25,30].

Following a long period of dominance of the Delta variant, in November 2021, another variant of concern has emerged in South Africa and Botswana—BA.1/B.1.1.529 also designated Omicron. Omicron has since then quickly over-powered Delta SARS-CoV-2. Its genome harbors 59 mutations, 36 of them are located within the spike, and 15 specifically within the RBD. Like Alpha, Omicron carries a P681H mutation within its FCS region. Recent reports show that Omicron partially escapes immunity elicited by infection or vaccines, with boosted individuals presenting waned vaccine efficacy over time. These reports also show that, compared to Delta, Omicron results in lower incidences of severe disease [21,23,39,40,41,42,43,44].

Here we used pseudoviruses to monitor the effects of FCS-spike mutations of either Alpha/Omicron (P681H), Beta (A701V), or Delta (P681R) on viral infectivity and neutralization sensitivity against sera that was drawn from fully vaccinated individuals. Single mutations within the FCS region were inserted to the wild-type Wuhan spike, as we demonstrated that single, P681H, V701A, and P681R FCS mutations have no role in viral infectivity or neutralization sensitivity. However, adding a single N501Y-RBD mutation into the wild type spike carrying FCS mutations substantially enhanced viral infectivity relative to wild-type SARS-CoV-2, with no effect on neutralization sensitivity. Similarly, adding the E484K-RBD mutation to pseudoviruses that carry an FCS-mutation decreased neutralization sensitivity against our tested sera, with no effects on viral infectivity.

We also tested the role of single mutations within Delta RBD-L452R, T478K, and the double L452R/T478K—on pseudovirus infectivity and neutralization sensitivity. Our analysis shows that, similarly to Delta pseudoviruses that carry L452R, T478K, and P681R, pseudoviruses with single L452R or K478T RBB mutations display a moderate reduction in neutralization sensitivity relative to wild-type SARS-CoV-2. In addition, viral infectivity of Delta SARS-CoV-2 pseudovirus is increased ×2-fold, while its single RBD mutations, L452R, and T478K or double L452R/T478K has no effect on infectivity levels. We employed this functional analysis approach by introducing RBD mutations that appear within the spike of lota SARS-CoV-2. Lota carries S477N/E484K mutations within its RBD, as well as a A701V-FCS mutation. Our analysis shows that the single S477N-RBD mutation has no impact on viral infectivity or neutralizing sensitivity to post vaccination sera. However, combining the RBD-N501Y mutation to S477N spike, enhances pseudoviral infectivity. Furthermore, inserting the E484K mutation into the S477N spike reduces the neutralizing sensitivity of these pseudoviruses to post vaccinated sera, with no effects on viral infectivity. We therefore conclude that, while FCS spike mutations have no impact on viral infectivity and neutralization potential, distinct RBD mutations respectively drive viral infectivity and neutralization sensitivity to post vaccination sera of SARS-CoV-2 variants. Furthermore, we validate the efficacy of the Pfizer third dose vaccine against Delta and Lota SARS-CoV-2 variants.

## 2. Materials and Methods

### 2.1. Lead Contact

Resources and reagents generated in this study are available from the Lead Contact with completed Materials Transfer Agreement. Further information and requests for resources and reagents should be directed to the Lead Contact, and will be fulfilled by the lead contact, Ran Taube (rantaube@bgu.ac.il).

### 2.2. Human Subject Collection

The study was conducted in compliance with ethical principles of the Declaration of Helsinki and approved by the Soroka Medical Center Institutional Review Board (protocol 0281-20-SOR). Sera was collected from a cohort of 10 individuals that were vaccinated in the first or second Pfizer vaccine. Samples were from either a 21-day post first dose, or 9–11 days post second dose. 

### 2.3. Bacterial Strains and Cell Culture

HEK-ACE2 stable cells were cultured at 37 °C in a 5% CO_2_ incubator. Cells were grown in Dulbecco’s Modified Eagle Medium (DMEM) high glucose (Gibco, Waltham, MA, USA), supplemented with 10% fetal bovine serum (FBS), 2 mM GlutaMAX (Gibco), and 100 U/mL penicillin-streptomycin. HEK-ACE2 expressing cells were generated by stable transduction with lentivirus expressing human ACE2. Our pseudoviruses were standardized for equal loads by monitoring p24 levels by ELISA. *E. coli* DH5 bacteria were used for transformation of plasmids coding for lentivirus packaging DNA and SARS-CoV-2 spike. A single colony was picked and cultured in LB broth with 50 g penicillin at 37 °C at 200 rpm in a shaker for overnight.

### 2.4. Generation of HEK-hACE2 Stable Cell Line

hACE2 (received from S. Pohlmann lab, University of Göttingen, Gottingen, Germany) was re-cloned into a lentiviral expression vector. Lentiviral particles were produced as described previously [45,46]. Briefly, HEK293T cells were stably transduced with lentivirus expressing ACE2. Cells were analyzed for hACE2 expression by FACS, using a biotinylated-labeled spike (ACROBiosystems. Newark, DE, USA). High ACE2 expressing cells were sorted using FACS Aria. ACE2 expression was periodically monitored by FACS. For TMPRSS2 expression, HEK-ACE2 cells were transfected with an expression plasmid of the protease 48 h before infection. Protein expression was verified by Western blotting upon harvesting SARS-CoV-2 infected cells.

### 2.5. Construction of Spike Mutants

A QuikChange Lightening Site-Directed Mutagenesis kit was used to generate amino acid substitutions in the pCDNA wild-type spike plasmid carrying a D614G (received from S. Pohlmann lab, University of Göttingen, Göttingen, Germany), following the manufacturer’s instructions (Agilent Technologies, Inc., Santa Clara, CA, USA). For each mutant, the relative oligos that harbored the required mutation were employed. 

### 2.6. Generation of Pseudotyped Lentivirus and Neutralization Assays

Pseudotyped viruses were generated in HEK293T cells. Briefly, LTR-PGK luciferase lentivector was transfected into cells together with other lentiviral packaging plasmids coding for Gag, Pol Tat Rev, and the corresponding wild-type or mutate spike envelopes. Transfections were done in a 10 cm format, as previously described and supernatant containing virus were harvested 72 h post transfection, filtered and stored at −80 °C [46]. Neutralization assays were performed with pseudoviruses expressing different spike proteins. Equal levels of viruses used for neutralization and infectivity assays were obtained following measurements of p24 levels using p24 enzyme-linked immunosorbent assay (ELISA) for the presence of HIV-p24 antigen as described [47]. Neutralization assays were performed in a 96-well format, in the presence of pseudotyped viruses that were incubated with increasing dilutions of the tested sera (1:2000; 1:8000; 1:32,000; 1:128,000) or without sera as a control. Cell-sera were for 1 h at 37 °C, followed by transduction of HEK-ACE2 cells for additional 12 h. In addition, 72 h post transduction, cells were harvested and analyzed for luciferase readouts according to the manufacturer protocol (Promega). Neutralization measurements were performed in triplicates using an automated Tecan liquid handler and readout were used to calculate NT_50_-50% inhibitory titers concentration.

### 2.7. Pseudoviruses Quality Control and Tittering

To determine the titers of pseudoviruses, 1e5 ACE2 stable HEK cells were plated in a 12-well plate. In addition, 24 h later, decreased serial dilutions of pseudovirus were used to transduce cells. Furthermore, 48 h post transduction, cells were harvested and analyzed for their luciferase readouts. p24 ELISA measurements were conducted to ensure equal loads. 

### 2.8. Quantification and Statistical Analysis

Statistical analyses were performed using GraphPad Prism. Measured statistical significance was calculated between experiments by a two-tailed Student’s *t* test—*p* ≤ 0.001. Error bars throughout all figures represent one standard deviation. Specific details on statistical tests and experimental replicates can be found in the figure legends.

## 3. Results

### 3.1. Infectivity Levels and Neutralizing Potential of Post-Vaccination Sera against Pseudoviruses Carrying Single FCS Spike Mutations

We acquired blood samples from a cohort of post-vaccination individuals who received three doses (10–14 days post third dose; *n* = 20) of the BNT162b2-Pfizer vaccine and assessed their potential to neutralize infection of SARS-CoV-2 pseudoviruses of HEK-target cells that express the human ACE2 receptor. Neutralization potential of these samples was monitored against pseudoviruses that harbored mutations within the Furin-Cleavage Site (FCS) or RBD of spike. 

**Figure 1:**
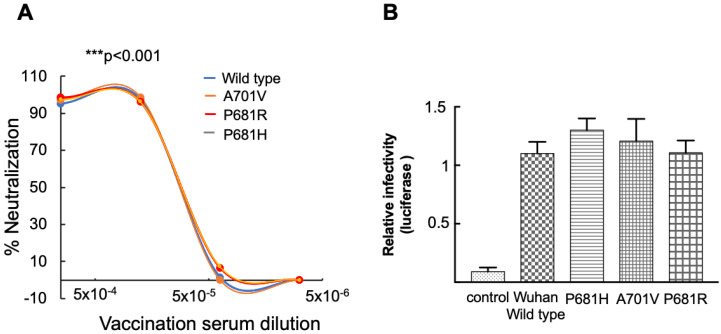
Furin Cleavage Site spike mutations have no impact on neutralizing potency against post-vaccination sera and on viral infectivity. (**A**) Neutralizing potential of pseudoviruses that carry Furin Cleavage Site mutations against post-vaccination sera-neutralization assays were performed by transducing HEK293T-ACE2 cells with a pseudovirus displaying a wild-type SARS-CoV-2 spike or its FCS-P681H V710A P681R mutants, in the presence of increasing dilutions of sera drawn from post vaccinated individuals. In addition, 48 h post transduction, cells were harvested, and their luciferase readings were monitored. Neutralizing potency was calculated at increased serial dilutions, relative to transduced cells with no sera added. Neutralization, NT_50_, is defined as the inverse dilution that achieved 50% neutralization. Results are the average of two independent biological experiments. Triplicates were performed for each tested serum dilution. Black bars represent geometric mean of NT_50_ values, indicated at the top. Statistical significance was determined using a one tailed *t*-test *** < 0.001; (**B**) infectivity levels of pseudoviruses carrying Furin Cleavage Site mutations. Pseudoviruses bearing wild-type SARS-CoV-2 spike or the indicated FCS-P681H, A701V, and P681R spike mutants were used to transduce HEK293T-ACE2 cells. Equal viral loads were normalized based on p24 protein levels. In addition, 48 h post transduction, cells were harvested, and their luciferase readouts were monitored. Bar graphs show mean values ± SD error bars of three independent experiments.

Initially, single point FCS mutations were inserted into the Wuhan wild-type (D614G) spike. These included either Alpha (P681H), Beta (A701V), or Delta (P681R) mutations. Omicron spike also exhibits a P681H-FCS mutation and was tested as well. Our findings show that pseudoviruses carrying each of these single FCS mutations at position P681 were similarly neutralized by post-vaccination sera as wild-type SARS-CoV-2 pseudoviruses (Figure 1A).

We next monitored infectivity levels of our pseudoviruses by testing their ability to infect HEK-ACE2 target cells (Figure 1B). As our experimental platform used single-round pseudoviruses, the term transduction is more suitable than infectivity that would imply the use of infections SARS-CoV-2. However, a close correlation exists between pseudoviruses and live viruses regarding measurements of viral entry infection and neutralization potential [20,47,48,49,50,51,52,53]. Our findings demonstrate that pseudoviruses carrying either Alpha-FCS-P681H mutation, Beta-A701V, or Delta-P681R efficiently transduce their target cells, and infectivity levels were similar to the levels exhibited by pseudoviruses that carried Wuhan wild-type P681 spike. Importantly, the Wuhan pseudovirus could not transduce control cells that did not express ACE2. We conclude that FCS spike mutations P681H, A701V, and P681R have no impact on viral infectivity or vaccine-elicited neutralization potential (Figure 1B).

Additionally, as the FCS region has been suggested to play an important role in TMPRSS2-mediated viral infection and fusion between the virus and its target cells, we tested the effects of TMPRSS2 host protease in pseudovirus infection. TMPRSS2 has been suggested to play a key role in enhancing SARS-CoV-2 infectivity by promoting S2 cleavage [23,34]. HEK-ACE2 engineered cells were transduced by the Wuhan pseudovirus, and viral transduction was monitored. Our data show that TMPRSS2 had no effects on pseudovirus infectivity, exhibiting similar levels of infection upon expression or absence of TMPRSS2 (Figure S1).

### 3.2. N501Y RBD Mutation Enhances Viral Infectivity of Pseudoviruses Carrying FCS Spike Mutation

**Figure 2:**
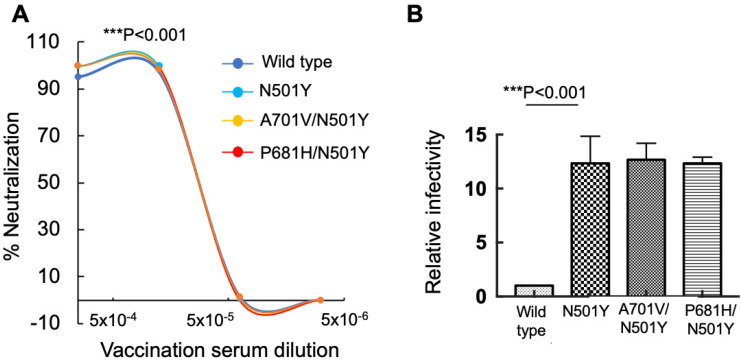
N501Y RBD mutation drives viral infectivity of pseudoviruses carrying Furin Cleavage Site mutations. (**A**) N501Y RBD mutation has no impact on neutralization sensitivity of SARS-CoV-2 that carry a Furin Cleavage Site against post vaccination sera. Neutralization assays were performed by transducing HEK293T-ACE2 cells with pseudovirus displaying a wild-type SARS-CoV-2 spike. Single N501Y mutation or FCS-P681H/N501Y or A701V/N501Y combined mutations in the presence of increasing dilutions of sera drawn from post-vaccinated individuals. In addition, 48 h post transduction, cells were harvested and their luciferase readings were monitored. Neutralizing potency was calculated at increased serial dilutions, relative to transduced cells with no sera added. Neutralization, NT_50_, is defined as the inverse dilution that achieved 50% neutralization. Results are the average of two independent biological experiments. Triplicates were performed for each tested serum dilution. Black bars represent geometric mean of NT_50_ values, indicated at the top. Statistical significance was determined using a one tailed *t*-test *** < 0.001. (**B**) N501Y RBD mutation enhances viral infectivity of SARS-CoV-2 that carry a Furin Cleavage Site. Pseudoviruses carrying wild-type SARS-CoV-2 spike, single N501Y, or the double P681H/N501Y or A701V/N501Y mutations were used to transduce HEK293T-ACE2 cells. Equal viral loads were normalized based on p24 protein levels. In addition, 48 h post transduction, cells were harvested and their luciferase readouts were monitored. Bar graphs show mean values ± SD error bars of three independent experiments. Measured statistical significance was calculated between experiments by a two-tailed Student’s *t* test *** *p* ≤ 0.001.

We further generated pseudoviruses that carried in addition to a unique FCS-P681H or A701V mutations, a single N501Y-RBD mutation within the wild-type spike. Previous reports show that the N501Y mutation increases affinity of the viral spike to its ACE2 human receptor [48,54]. We then monitored neutralization sensitivity of our engineered pseudoviruses to the post vaccination sera (Figure 2A). Infectivity levels were also tested and compared to those of pseudoviruses that carried a wild-type Wuhan spike (Figure 2B). We show that a single N501Y-RBD mutation had no effect on neutralization sensitivity against post vaccinated sera, exhibiting levels similar to wild-type pseudoviruses (Figure 2A). Moreover, pseudoviruses that carried double-P681H/N501Y, or A701V/N501Y mutations within their wild-type spike were also tested and exhibited similar neutralization potential as the single mutated N501Y pseudoviruses, or wild-type SARS-CoV-2 pseudoviruses (Figure 2A). Overall, these data conclude that the N501Y does not play a role in promoting antibody escape and neutralization. 

We next monitored the infectivity levels of the single FCS-N501Y pseudoviruses and the double P681H/N501Y or A701V/N501Y pseudoviruses (Figure 2B). Our analysis confirmed previous results showing that N501Y pseudoviruses exhibited higher levels of viral infectivity that were ×11-fold higher relative to wild-type SARS-CoV-2 pseudoviruses [20,49,55]. Moreover, pseudoviruses that carried the A7101V/N501Y or P681H/N501Y double mutations also presented enhanced infectivity levels, which were ×11–12-fold higher relative to the wild-type SARS-CoV-2 or pseudoviruses that carried single P681H, and A701V-FCS mutations (refer to Figure 1B). We therefore conclude that the RBD-N501Y mutation is solely responsible for enhanced viral infectivity detected in SARS-CoV-2 variants that carry this mutation.

### 3.3. E484K RBD Mutation Moderately Reduces Neutralization Sensitivity of Pseudoviruses Carrying FCS Spike Mutation against Post-Vaccinated Sera

**Figure 3:**
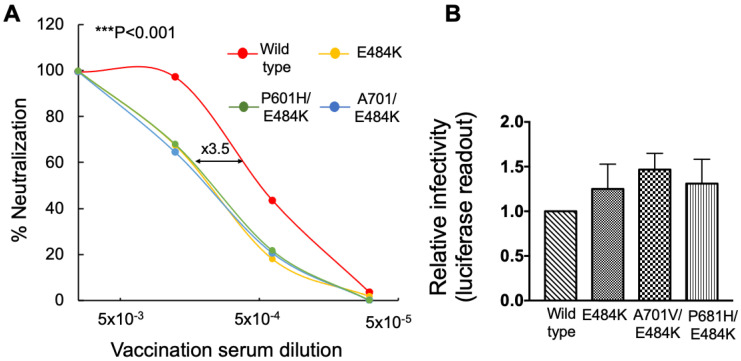
E484K RBD mutation reduces neutralization sensitivity of pseudoviruses carrying Furin Cleavage Site spike mutations against post-vaccination sera. (**A**) E484K spike mutation reduces neutralization sensitivity of pseudoviruses that carry a Furin Cleavage Site against post-vaccination sera. Neutralization assays were performed by transducing HEK293T-ACE2 cells with pseudovirus displaying a wild-type SARS-CoV-2 spike or its FCS-P681H/E484K or V710A/E484K combined mutants, in the presence of increasing dilutions of sera drawn from post vaccinated individuals. In addition, 48 h post transduction, cells were harvested and their luciferase readings were monitored. Neutralizing potency was calculated at increased serial dilutions, relative to transduced cells with no sera added. Neutralization, NT_50_, is defined as the inverse dilution that achieved 50% neutralization. Results are the average of two independent biological experiments. Triplicates were performed for each tested serum dilution. Black bars represent geometric mean of NT_50_ values, indicated at the top. Statistical significance was determined using one tailed *t*-test *** < 0.001. (**B**) E484K RBD mutation has a moderate impact on infectivity levels of SARS-CoV-2 Furin Cleavage Site spike mutation-Pseudoviruses bearing wild-type SARS-CoV-2 spike or the indicated P681H/E484K or A701V/E484K combined mutants were used to transduce HEK293T-ACE2 cells. Equal viral loads were normalized based on p24 protein levels. Furthermore, 48 h post transduction, cells were harvested and their luciferase readouts were monitored. Bar graphs show mean values ± SD error bars of three independent experiments.

We further generated pseudoviruses that carried single E484K RBD mutation, as well as double mutated pseudoviruses that carried P681H/E484K, or A701V/E484K mutations, and tested their neutralization sensitivity against our post-vaccination cohort. Previous reports show that E484K is critical for increased neutralization resistance of SARS-CoV-2 variants [20,48,56,57]. Our data confirmed that pseudoviruses with a single RBD-E484K mutation exhibited a moderate decrease in neutralization sensitivity to post vaccination sera (1, 2) (Figure 3A). Moreover, pseudoviruses that carried the double P681H/E484K, or A701V/E484K mutations also presented decreased sensitivity of ×3.5-fold relative to wild-type SARS-CoV-2 pseudoviruses (Figure 3A).

When analyzing the infectivity levels of the pseudoviruses, with either P681H/E484K or A701V/E484K spike mutations, these exhibited a moderate increase in infectivity levels which was ×1.5-fold higher than a wild-type or single E484K SARS-CoV-2 pseudovirus (Figure 3B). We therefore conclude that the E484K mutation in a spike is mainly important for enhanced neutralization potential, with low effects on viral infectivity.

### 3.4. The Role of Specific RBD Mutations within the Spike of Delta SARS-CoV-2 on Neutralization Potential and Viral Infectivity

**Figure 4:**
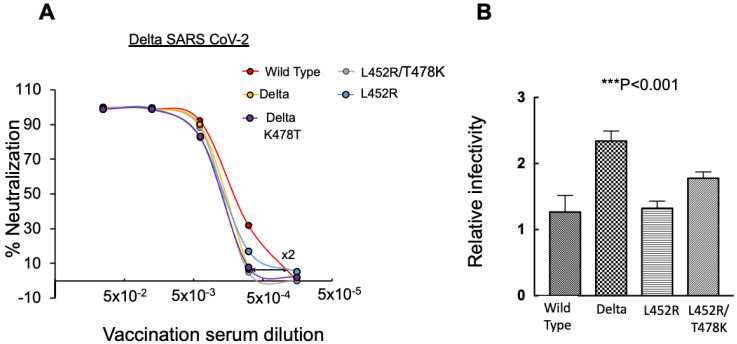
Infectivity levels and neutralization sensitivity of Delta SARS-CoV-2 and its unique RBD mutations. (**A**) Specific RBD residues of Delta spike that determine neutralization sensitivity to post vaccination sera-Neutralization assays were performed by transducing HEK293T-ACE2 cells with pseudoviruses displaying a wild-type SARS-CoV-2 spike or its specific Delta spike RBD single mutations, L452R, or T478K and double L452R/T478K. In addition, 48 h post transduction, cells were harvested and their luciferase readings were monitored. Neutralizing potency was calculated at increased serial dilutions, relative to transduced cells with no sera added. Neutralization, NT_50_, is defined as the inverse dilution that achieved 50% neutralization. Results are the average of two independent biological experiments. Triplicates were performed for each tested serum dilution. Black bars represent geometric mean of NT_50_ values, indicated at the top. Statistical significance was determined using a one tailed *t*-test *** < 0.001. (**B**) Delta RBD spike mutations impact infectivity levels of SARS-CoV-2-Pseudoviruses bearing wild-type SARS-CoV-2 spike or the indicated Delta RBD spike mutations. Specific indicated pseudoviruses were used to transduce HEK293T-ACE2 cells. Equal viral loads were normalized based on p24 protein levels. In addition, 48 h post transduction, cells were harvested and their luciferase readouts were monitored. Bar graphs show mean values ± SD error bars of three independent experiments.

To further evaluate the functional significance of spike RBD mutations in neutralization potential and viral infectivity, we generated pseudoviruses that exhibited within their spike specific mutations that appear within Delta SARS-CoV-2. The Delta variant of concern was initially identified in India and has become dominant before Omicron emerged and took over. The Delta spike carries unique RBD mutations including L452R, T478K, and P681R. We initially tested neutralization sensitivity of Delta pseudoviruses against post-vaccinated sera and demonstrated a reduced ×2-fold decrease in their ability to neutralize post-vaccinated sera (Figure 4A). These results align with our previous results showing a modest reduction in Delta neutralization sensitivity to post vaccinated sera following the second dose [58]. Additionally, single RBD mutations were also introduced into the wild-type spike and tested for effects on neutralization potential and infectivity levels employing pseudotyped infectivity and neutralization assays. We showed that pseudoviruses that carried a single L452R RBD mutation exhibited a slight reduction in neutralization potential of about ×1.5-fold relative to the wild-type SARS-CoV-2 pseudovirus (Figure 4A). Furthermore, pseudoviruses that carried the single T478K RBD mutation similarly showed a moderate ×1.7-fold reduction in their neutralization sensitivity relative to wild-type pseudoviruses. Finally, neutralizing potential of pseudoviruses that carried the double L452R/K478T-RBD mutations also exhibited a reduced ×1.9-fold neutralizing potential relative to the wild-type SARS-CoV-2. We therefore conclude that both T478K and L452R mutations equally contribute to the neutralizing sensitivity of Delta SARS-CoV-2 and overall, the reduction of neutralization potential and viral infectivity is moderate relative to wild-type pseudoviruses.

We then tested the infectivity levels of our engineered pseudoviruses. While Delta pseudoviruses exhibited a ×2-fold increase in their viral infectivity relative to the Wuhan wild-type SARS-CoV-2 pseudovirus, the single L452R mutated pseudovirus had no effects on viral infectivity. Furthermore, the double L452R/T478K pseudoviruses exhibited only moderate enhancement in infectivity levels (×1.2-fold) relative to wild-type SARS-CoV-2 (Figure 4B). 

We thus conclude that L452R and T478K RBD mutations play a role in the moderate decrease in the ability of Delta to neutralize post vaccination sera or in its infectivity. 

### 3.5. N501Y and E484K-RBD Mutations Respectively Drive Neutralization Potential and Infectivity of the Lota Variant

**Figure 5:**
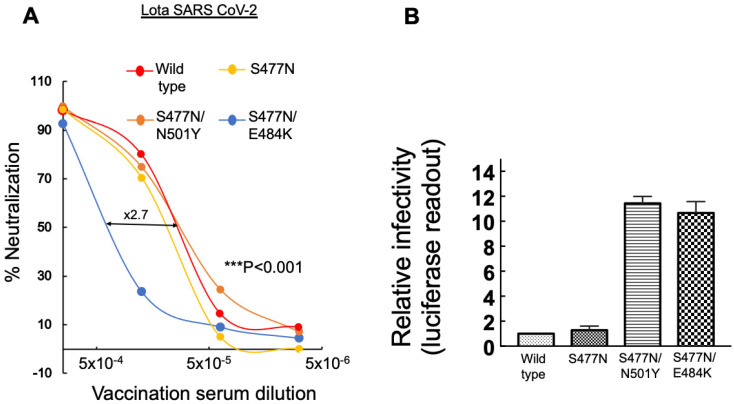
Infectivity levels and neutralization sensitivity of lota-SARS-CoV-2 variant of interest. (**A**) Effects of N501Y and E484K-RBD mutations on neutralizing potential of post-vaccination sera against pseudoviruses displaying S477N spike mutation-neutralization assays were performed by transducing HEK293T-ACE2 cells with a pseudovirus displaying a wild-type SARS-CoV-2 spike carrying B.1.526 (S477S) mutant, in the presence of increasing dilutions of sera drawn from post vaccinated individuals. In addition, 48 h post transduction, cells were harvested and their luciferase readings were monitored. Neutralizing potency was calculated at increased serial dilutions, relative to transduced cells with no sera added. Neutralization, NT_50_, is defined as the inverse dilution that achieved 50% neutralization. Results are the average of two independent biological experiments. Triplicates were performed for each tested serum dilution. Black bars represent a geometric mean of NT_50_ values, indicated at the top. Statistical significance was determined using a one tailed *t*-test *** < 0.001. (**B**) Effects of N501Y and E484K RBD of mutations on infectivity levels of pseudoviruses displaying S477N spike mutation-pseudoviruses bearing wild-type or lota-B.1.526 SARS-CoV-2 spike mutations (S477S/E484K) or its single S477N spike mutants were used to transduce HEK-ACE2 cells. Equal viral loads were normalized based on p24 protein levels. Furthermore, 48 h post transduction, cells were harvested and their luciferase readouts were monitored. Bar graphs show mean values ± SD error bars of three independent experiments. Measured statistical significance was calculated between experiments by a two-tailed Student’s *t*-test *** *p* ≤ 0.001.

To further evaluate the functional significance of RBD mutations, we employed a similar functional approach on pseudoviruses that carry spike mutations that correspond to the lota-B.1.526 variant (Figure 5). Lota SARS-CoV-2 emerged in New York and exhibits unique S477N and E484K RBD mutations within its spike, as well as A701V-FCS mutation. However, unlike Delta, it did not spread.

We initially tested neutralization sensitivity of pseudoviruses that carried a single S477N spike RBD mutation against our post-vaccinated sera. We showed that S477N-pseudoviruses exhibited an insignificant reduction of neutralization potential relative to wild-type SARS-CoV-2-×1.2 fold (Figure 5A). Upon monitoring the infectivity levels of single S477N pseudoviruses, we showed that infectivity levels of these engineered pseudoviruses exhibited similarly to those observed for wild-type SARS-CoV-2 (Figure 5B).

We next tested the impact of adding N501Y-RBD mutation to pseudoviruses that already carried S477N-RBD mutation (Figure 5A). Our analysis determined that the double S477N/N501Y mutations had no substantial impact on neutralization sensitivity, which was similar to the wild-type SARS-CoV-2 and to pseudoviruses that carried S477N RBD mutation (Figure 5A). However, monitoring infectivity levels of pseudoviruses that carried the double S477N/N501Y exhibited a ×12-fold increase in viral infectivity relative to wild type or single S477N pseudoviruses (Figure 5B). These results confirm the critical importance of the N501Y-RBD mutation for enhancement of viral infectivity.

We next monitored the neutralization sensitivity and infectivity of pseudoviruses that carried double S477N/E484K-RBD spike mutations. We documented a ×2.7-fold decrease in neutralization sensitivity relative to wild-type pseudoviruses, again showing the significant role of E484K in antibody escape of SARS-CoV-2 (Figure 5A). Moreover, the double S477N/E484K mutation pseudoviruses also exhibited high infectivity levels relative to wild-type SARS-CoV-2 pseudoviruses, presenting ×10-fold increase in infectivity levels, relative to wild type SARS-CoV-2 (Figure 5B). We conclude that E484K RBD mutation plays a key role in antibody escape of SARS-CoV-2 and leads to reduction in neutralization sensitivity. Moreover, in combination with S477N, it enhances viral infectivity.

## 4. Discussion

Currently administrated vaccines that have been developed in a relatively short time frame have successfully limited the COVID-19 pandemic, reducing viral spread and limiting hospitalization. These vaccines target the spike of SARS-CoV-2 and efficiently induce the development of neutralizing antibodies that inhibit viral infection. Despite these achievements, the overwhelming spread of the virus constantly drives the emergence of new viral variants that resist vaccine-induced neutralization. Alpha, Beta, Delta, and Omicron variants all carry numerous mutations in their spike, specifically within the RBD, which overall enhance viral transmission and mediate escape from vaccine-elicited or monoclonal neutralizing antibodies. As such, these variants were defined variants of interest or concern by the WHO, as they potentially compromise vaccine efficiency. 

Along RBD mutations that are extensively studied for their effects on viral evasion against vaccine-elicited antibodies, circulating variants also carry mutations within their polybasic Furin Cleavage site (FCS). FCS mutations include P681H in Alpha and Omicron, A701V in Beta, and P681R in Delta—indicating that the P681 residue plays a critical role in viral fitness. However, effects of FCS mutations on viral infectivity and neutralization escape have not been thoroughly investigated and their combined contribution to RBD mutations has also not been employed.

In this study, we used pseudoviruses that express spike proteins from different SARS-CoV-2 variants to monitor the effects of mutations within FCS of either Alpha and Omicron (P681H), Beta and P1 (A701V), and Delta (P681R) on viral infectivity and neutralization sensitivity to post-vaccination sera drawn from fully vaccinated individuals. Our study has few limitations as it relies on our conclusions on pseudoviruses and not live viruses. However, our questions focus only on the first step of the virus life cycle-attachment of the viral particle to the ACE2 cell receptor and entry into the target cells-where pseudoviruses are a suitable model. Moreover, pseudoviuses are lentiviral-based single round replication viruses, and, as such, with no ability to extend their use into late stages of virus production and release. Nevertheless, the pseudovirus system has been broadly tested in the literature for measuring vaccine neutralization efficiency against SARS-CoV-2. Numerous studies have demonstrated high correlation between vaccine-neutralization titers measured against pseudovirus and live SARS-CoV-2 [20,47,48,49,50,51,52,53]. Additionally, it is worth stating that our findings are relevant only to the tested sera. Another limitation is that in our system viral infection occurs in a non-clinical cell system, and therefore does not exhibit dependency in TMPRSS2, which has been shown to be required for viral cell entry on the surface of the target cell and for fusion between viral and cell membranes.

We show that FCS mutations within a spike have no impact on either neutralization sensitivity or viral infectivity (Figure 1). However, upon adding an N501Y RBD mutation, infectivity levels of FCS-exhibiting pseudoviruses were enhanced relative to wild-type SARS-CoV-2. In contrast, neutralization sensitivity of single or double FCS/N501Y pseudoviruses against post-vaccinated sera remained similar to wild-type SARS-CoV-2 pseudoviruses (Figure 2). Adding a E484K-RBD mutation to FCS-pseudoviruses, moderately decreased neutralization sensitivity against our sera samples, with no effects on viral infectivity. Overall, we conclude that RBD mutations-N501Y and E484K dictate the ability of the virus to efficiently spread, by respectively modulating viral infectivity and ability to resist the humoral response that is induced by vaccination. These results are in agreement with previous work that emphasized the key role of RBD mutations in viral infection and neutralizing antibodies escape [15,59]. These reports demonstrate that spike residues like D614G and N501Y are located at the distal region of the spike RBD and facilitate transitions from a closed to open state of the spike prior to ACE2 binding, enhancing the stability and affinity of the viral spike to its receptor and subsequently affecting viral spread [16,60].

It is important to note that our study relates only to the effects of FCS mutations on viral infectivity and to post-vaccination neutralization response in a pseudovirus system. Recent work has confirmed that the basic PRRAR motif in FCS is important for viral transmission, as it provides an advantage for the virus to enter its target cells at the cell surface, promoting membrane fusion between the virus and the cell, primarily in lung and in human epithelial cells [38,61]. In this scenario, the virus successfully escapes the innate IFITM2 response upon entry and supports efficient transmission [25,30]. On the other hand, in viruses that lack the FCS, viral entry is mediated through endosomes, thus being exposed to the IFITM2- innate response. In Vero E6 cells, which do not express TMPRSS2 protease, SARS-CoV-2 deleted FCS gains an advantage, potentially because there is an increase in its spike stability, as spike cleavage may result in premature shedding of the S1 subunit and abrogates receptor binding [62]. Knockout of TMPRSS2 or deletion of FCS sequences also resulted in impaired infection, which was due to low viral titers that were shed from an infected ferrets animal model, resulting in reduced transmission to cohoused sentinel animals [25]. Moreover, given the fact that, upon propagating the virus in cell culture, FCS sequence is lost or mutated, while, in infectious viral isolates, these mutations are at a very low level, further strengthening the assumption that an FCS sequence is critical for viral transmission, only in clinically relevant systems [25]. In another recent work, the significance of the FCS was further reinforced. FCS-P681R mutation within the Kappa and Delta SARS-CoV-2 variants augmented syncytium formation, thus contributing to increased infectivity of the UK and SA variants [63]. However, in our study, we could not see such an effect on viral infection as our infectivity assays used single round pseudoviruses and focused only on the early step of entry and infection, with no ability to extend the effects on late stages of virus release. Moreover, we did not perform our assays in a clinically relevant cells, where viral infection is dependent on TMPRSS2 and supports membrane fusion to facilitate syncytium formation [63].

We also determined the effects of RBD mutations that appear within the spike of the Delta SARS-CoV-2 variant of concern. Single L452R and T478K RBD mutations moderately impact the neutralization potential, reaching about ×2 fold reduction (Figure 4). The same results were obtained when there was a double L452R/T478K RBD mutation. These results are comparable to the Delta SARS-CoV-2 that carries the complete, L452R/T478K/P681R-Delta RBD mutations. These results align with our previous data on Delta SARS-CoV-2 [58] and state that both L452R and T478K combine to equally affect the moderate decrease in neutralizing sensitivity and viral infectivity that is exhibited by Delta SARS-CoV-2 (Figure 4). Nevertheless, other mutations outside the spike may also be ‘considered to affect viral neutralization sensitivity and infectivity.

The significance of RBD mutations was also demonstrated with the Lota SARS-CoV-2 that carries S477N/E484K within its spike. Our data showed that, while S477N-RBD mutation alone had no impact on both viral infectivity or neutralizing sensitivity to post vaccination sera, the addition of N501Y or E484K spike mutations enhanced viral infectivity and decreased neutralizing sensitivity to post vaccinated sera (Figure 5).

In this work, only sera that drawn from vaccinated individuals who received the third dose of the Pfizer-BTN162b2 mRNA vaccine has been tested. However, several other vaccines, specifically Moderna mRNA-1273 and the Ad26 CoV2.S from Janssen/J&J, have been developed and successfully applied for immunization against SARS-CoV-2. These vaccines exhibit high efficiency in preventing viral infection and disease progression [10,13,21,64]. However, with the rapid spread of newly emerged variants and the waning of the immune response that is elicited by them, current vaccines lose their effectiveness and therefore lead to an urgent need to develop new versions [65,66,67]. Nevertheless, our results on the role of spike specific mutations will probably also apply for other vaccines versions that were not tested in this study. Therefore, as FCS mutations that are positioned away from the RBD region and do not play a role in ACE2 binding to spike, we reason that these will also play no role in viral neutralization by sera from individuals who were vaccinated by other vaccines. However, RBD-specific mutations like N501Y, E484K, S477N, and L452R K417N, which lay within or close to the binding surface to ACE2, will keep playing key roles in respectively affecting binding of the spike to the receptor or escaping from mAb.

Taken together, our results highlight the importance of specific residues within the RBD of spike in driving neutralization sensitivity and viral infectivity of different SARS-CoV-2 variants. N501Y L452R, E484K S477S, and T478K distinct RBD mutations potentially compromise the efficacy of administrated vaccines.

## Data Availability

Data are available from corresponding author upon request.

## Supplementary Materials

The following supporting information can be downloaded at: https://www.mdpi.com/article/10.3390/v14040803/s1, Figure S1: TMPRSS2 does play role in viral infectivity of pseudoviruses that infect HEK-293T-ACE2-TMPRSS2 cells.
