# Supplementary material for: Functional Analysis of Spike from SARS-CoV-2 Variants Reveals the Role of Distinct Mutations in Neutralization Potential and Viral Infectivity
Source: Viruses. 2022 Apr 13;14(4):803. doi: 10.3390/v14040803 (PMC9030214; doi:10.3390/v14040803)
Supplement: Supplementary file 1 [file viruses-14-00803-s001.zip › viruses-1647282-supplementary.pdf]

viruses

Supplementary Materials

Functional Analysis of Spike from SARS-CoV-2 Variants Reveals the Role of Distinct Mutations in Neutralization Potential and Viral Infectivity

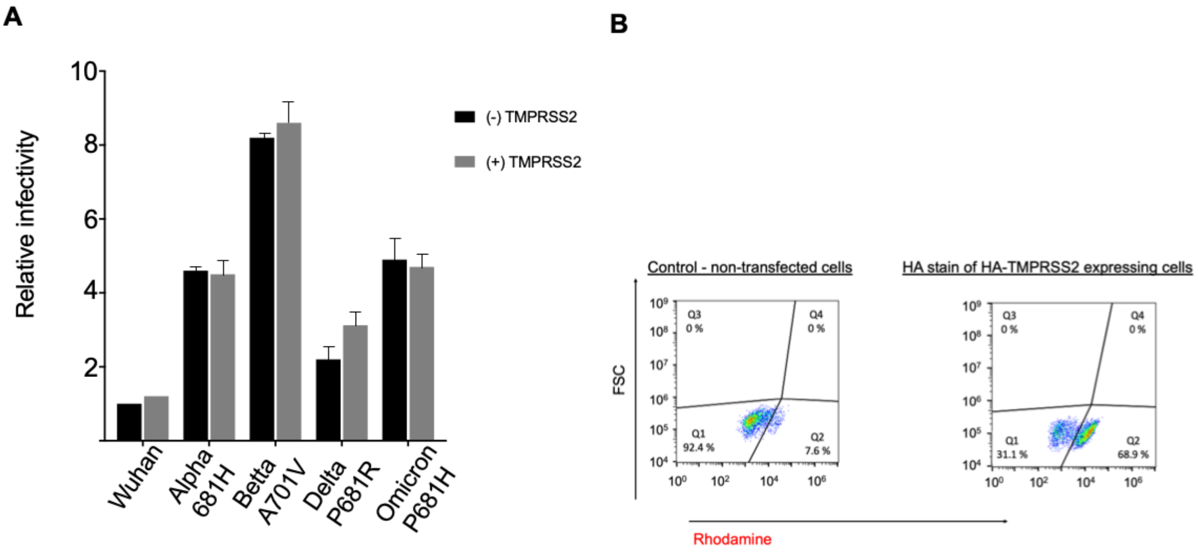

Figure S1. TMPRSS2 does play role in viral infectivity of pseudoviruses that infect HEK-293T-ACE2-TMPRSS2 cells.

(**A**) Pseudoviruses bearing the indicated SARS-CoV-2 spike mutations were used to transduce HEK-ACE2 cells and HEK cells that express TMPRSS2. The human protease was transfected into cells 48 hr earlier and its expression was verified in infected cells by FACS analysis (**B**). Equal viral loads were normalized based on p24 protein levels. 48 hr post transduction, cells were harvested and their luciferase readouts were monitored. Bar graphs show mean values ± SD error bars of three independent experiments. Measured statistical significance was calculated between experiments by a two-tailed Student's t test.
